# Blunted Cardiac Parasympathetic Activation in Student Athletes With a Remote History of Concussion: A Pilot Study

**DOI:** 10.3389/fneur.2020.547126

**Published:** 2020-09-30

**Authors:** Mohammad Nadir Haider, Blair D. Johnson, Emily C. Horn, John J. Leddy, Charles G. Wilber, Emma L. Reed, Morgan O'Leary, Adam Bloomfield, Larissa L. Decezaro, Barry S. Willer

**Affiliations:** ^1^UBMD Department of Orthopaedics and Sports Medicine, Jacobs School of Medicine and Biomedical Sciences, State University of New York at Buffalo, Buffalo, NY, United States; ^2^Department of Neuroscience, Jacobs School of Medicine and Biomedical Sciences, State University of New York at Buffalo, Buffalo, NY, United States; ^3^Department of Biostatistics, School of Public Health and Health Professions, State University of New York at Buffalo, Buffalo, NY, United States; ^4^Department of Exercise and Nutrition Sciences, School of Public Health and Health Professions, State University of New York at Buffalo, Buffalo, NY, United States; ^5^Jundiaí Medical School, Faculdade de Medicina de Jundiaí (FMJ), Jundiai, Brazil; ^6^Department of Psychiatry, Jacobs School of Medicine and Biomedical Sciences, State University of New York at Buffalo, Buffalo, NY, United States

**Keywords:** concussion, autonomic nervous system, sport, heart rate vaiability, face cooling

## Abstract

**Introduction:** Blunted cardiac autonomic nervous system (ANS) responses, quantified using heart rate variability (HRV), have been reported after sport-related concussion (SRC). Research suggests this persists beyond clinical recovery. This study compared cardiac parasympathetic responses in student athletes with a remote history of SRC (> 1-year ago, Concussion History: CH) with those who reported no lifetime history of SRC (Concussion Naïve: CN).

**Design:** Retrospective nested case-control.

**Setting:** University laboratory.

**Patients or Other Participants:** CH (*n* = 9, 18.3 ± 2 years, 44% male, median 2 years since injury) were student athletes with a remote history of concussion(s) from more than 1 year ago. CN (*n* = 21, 16.7 ± 3 years, 67% male) were student athletes with no lifetime history of concussion. Exclusion criteria included taking medications that could affect ANS function, history of concussion within the past year, persistent concussion symptoms, lifetime history of moderate to severe brain injury, and lifetime history of more than 3 concussions.

**Material and Methods:** Participants performed the Face Cooling (FC) test for 3-min after 10-min of supine rest while wearing a 3-lead electrocardiogram in a controlled environment.

**Outcome Measures:** Heart rate (HR), R-R interval (RRI), root mean square of the successive differences (RMSSD) of RRI, high frequency (HF) and low frequency to HF (LF:HF) ratios.

**Results:** At baseline, CH had a lower resting HR than CN (62.3 ± 11 bpm vs. 72.9 ± 12, *p* = 0.034). CH had a different HR response to FC than CN (+8.9% change from baseline in CH vs. −7.5% in CN, *p* = 0.010). CH also had a smaller RMSSD increase to FC than CN (+31.8% change from baseline in CH vs. +121.8% in CN, *p* = 0.048). There were no significant group differences over time in RRI (*p* = 0.106), HF (*p* = 0.550) or LF:HF ratio (*p* = 0.053).

**Conclusion:** Asymptomatic student athletes with a remote history of concussion had a blunted cardiac parasympathetic response to FC when compared with athletes with no lifetime history of concussion. These data suggest that an impaired autonomic response to a physiological stressor persists after clinical recovery from SRC for longer than previously reported.

## Introduction

Concussion, a subset of mild traumatic brain injury (mTBI), is a physiological, (1) metabolic, (2) and microstructural (3) insult to the brain resulting in non-specific somatic, cognitive, and emotional symptoms (4). There is no gold-standard method for diagnosing concussion, nor is there agreement on which measures need to normalize before beginning a return-to-play (RTP) strategy. The most recent International Concussion in Sport Group guidelines consider sport-related concussion (SRC) to be one of the most complex injuries in sports medicine to diagnose, assess, and manage; therefore, it is recommend that multi-modal clearance criteria be used for RTP decisions (5).

Emerging research indicates there is altered cardiovascular autonomic nervous system (ANS) function after concussion (6–9). Damage to the primary ANS control centers located in the brainstem following concussion has been confirmed by diffusion tensor imaging (10). Physiological research has shown that concussed subjects demonstrate reduced baroreflex sensitivity moving from supine to standing (11), and have reduced heart rate variability (HRV) at rest (12) and during exercise (12), which may reflect functional uncoupling of ANS control of cardiovascular function. Face Cooling (FC), i.e., cooling the forehead, eyes, and cheeks, stimulates the trigeminal nerve to evoke transient (~1–2 min) increases in cardiac parasympathetic activity followed by sympathetically-mediated increases in blood pressure (13). In a prior study (14), concussed college-aged athletes within 10 days of injury demonstrated a blunted cardiac parasympathetic response to FC when compared with healthy controls (who did not report having a concussion within the past year). The concussed group also demonstrated lower sympathetically-mediated increases in blood pressure during FC. In this regard, we have also shown that recently concussed college athletes have blunted increases in heart rate (HR) and blood pressure during the cold pressor test, which is a sympathetic stressor (15). Collectively, these data indicate that both branches of the ANS do not respond properly to physiological stressors following a concussion.

Clinical recovery from a concussion tends to occur within ~2 weeks in adults and by 3–4 weeks in adolescents (5). A recent systematic review, however, found that a myriad of physiological abnormalities are detectable for up to 1 month or more after the resolution of symptoms from concussion (16). It is not yet known when, or if, physiological function returns to baseline following a concussion, yet studies assessing the physiology of concussion recruit healthy controls with remote concussion histories. Additionally, a majority of studies have focused on sympathetic engagement (12, 17, 18). Hence, the purpose of this study was to determine the parasympathetic response to FC in asymptomatic student athletes with a remote history of concussion. For this, we retrospectively identified healthy student athletes who were initially recruited as healthy controls in previous studies (14, 19). The inclusion criteria for healthy controls in those studies included (1) not experiencing a concussion within the past year and (2) no more than 3 lifetime concussions. We separated these subjects into those who reported a remote history of concussion more than 1 year prior to testing (Concussion History: CH) and those who reported no lifetime history of concussion (Concussion Naïve: CN). We controlled for sex and age, which affect cardiac ANS tone (20). We hypothesized that CH participants would have a blunted cardiac parasympathetic response to FC vs. CN participants.

## Methods

This study was approved by the University at Buffalo IRB and conducted in accordance with the latest standards set forth by the Declaration of Helsinki. Athletic, healthy participants were recruited from local high school and college sport teams. The study was explained and consent was obtained. Parental consent/assent was obtained for all minors. On the day of the physiological assessment, participants completed a questionnaire that included demographics (including number of previous concussions), a Post-Concussion Symptom Scale (PCSS) (21), and current sport participation.

### Participants

Participants in both groups had been recruited as healthy controls in previous studies (14, 19). CH were healthy male and female high school or college-aged athletes with a remote history of a concussion that occurred more than 1 year ago. CN were healthy male and female high school or college-aged athletes who reported never having experienced a concussion. Ages for both groups ranged from 13 to 24 years. Only physician (or relevant clinician)-diagnosed concussions were included. Participants were excluded if they (1) had a history of more than 3 lifetime concussions (because this is associated with persistent impairments) (22); (2) had a history of moderate or severe traumatic brain injury; (3) were currently on medications that would affect ANS function, e.g., mood disorder (tricyclic antidepressants) and/or learning disorder medications (methylphenidate, amphetamine), or beta-blockers; (4) did not participate in at least one organized sport; and (5) had a symptom severity score of more than 7/132 on the PCSS (23).

### Experimental Approach

Participants were instructed to refrain from alcohol, caffeine, and exercise for 12 h and food for 2 h prior to their visit. Participants were instrumented with a 3-lead electrocardiogram (ECG) (DA100C, Biopac Systems, Goleta, CA) and assumed the supine position for 10 min in a quiet environment prior to FC. FC was performed by placing a pliable plastic bag filled with 2.5 L of ice water (~0°C) on the forehead, eyes, and cheeks for 3 min. Room temperature was controlled and ranged from 20 to 23°C and humidity was controlled between 15 and 25%. Participants were allowed to end the test early if it became too uncomfortable. The FC test is based on mammalian diving reflex physiology (24), and the complete protocol and methods of data processing/analysis have been published previously (14). A variety of individual and environmental factors are known to affect HRV (25). These factors were assumed to affect each group equally and were not controlled for. This is discussed further in the limitations section.

### Data and Statistical Analyses

ECG waveforms were analyzed using commercially available software (WinCPRS and Kubios HRV Software 5.0) with built-in tools for ECG clean-up, including a QRS detector, beat-to-beat analysis, and R-wave correction. ECG was visually inspected at the time of the experimental procedure and the first 5 min of supine rest was discarded. Baseline values were taken as the mean of minutes 6 and 7 of supine rest. HR, R-R interval (RRI), and root mean square of the successive differences (RMSSD) of RRI (26) were derived from the time domain while high frequency (HF) and the ratio of low frequency to HF (LF:HF) were derived from the frequency domain using Fast Fourier transformation (27). Mann-Whitney U-Test was used to test for group differences in age, height and weight. χ^2^-test was used for group differences in sex. Mean values for HR, RRI, RMSSD, HF and LF:HF with 95% confidence intervals (CI) were calculated at baseline and during each minute of FC and compared using the Mann-Whitney U-Test. The HR, RRI, RMSSD, HF and LF:HF percent change from baseline with 95% CI were also calculated. Mixed-models repeated measures ANCOVA with history of concussion as the grouping variable and sex (binary) and age (continuous) as covariates was used to assess for statistical differences in HR, RRI, RMSSD, HF and LF:HF change over time. Due to the pilot nature of this investigation, no *post-hoc* analysis for multiple comparisons was performed. A *p*-value of < 0.05 was considered significant. Statistical analyses were performed using SPSS Version 24 (Armonk, NY).

## Results

Thirty-four participants performed FC. Three participants did not complete all 3 min of FC and one participant's ECG had several artifacts on visual inspection and was discarded; hence, 30 participants were included in the analyses. Nine participants were categorized as CH and 21 participants as CN. CH's most recent concussion occurred a median of 2 years (interquartile range 1–3, range 1–8 years) prior to testing. Group demographics are presented in Table 1.

**Table 1:** Participant demographics.

	**Concussion History (*n* = 9)**	**Concussion Naïve (*n* = 21)**	***p*-value**
Age (years)	18.3 ± 2.4	16.7 ± 3.0	0.162
Sex	4 males (44%)	14 males (67%)	0.255
Height (cm)	171 ± 11	172 ± 8	0.774
Weight (kg)	68.8 ± 15	66.1 ± 18	0.702
Previous concussion			–
1	6	-	
2	2	-	
3	1	-	

*Data are presented as mean ± standard deviations*.

Absolute mean HR, RRI, RMSSD, HF and LF:HF at baseline and during each minute of FC are presented in Table 2. CH had lower HR at baseline and higher LF:HF ratio at minute 1 vs. CN. No other differences for absolute values were observed between groups.

**Table 2:** Absolute values for mean heart rate, RRI, RMSSD, HF, and LF:HF ratio during FC.

	**Concussion History (*n* = 9)**	**Concussion Naïve (*n* = 21)**	***p*-value**
**HR in beats per minute**			
Baseline	62.3 ± 10.9	72.9 ± 12.2	**0.025**
Minute 1	66.6 ± 11.6	66.7 ± 10.9	0.689
Minute 2	70.1 ± 15.0	64.8 ± 10.9	0.209
Minute 3	65.2 ± 10.4	66.6 ± 12.4	0.859
**RRI in milliseconds**			
Baseline	908.9 ± 202	916.3 ± 71	0.926
Minute 1	916.5 ± 167	1016.1 ± 195	0.233
Minute 2	971.5 ± 198	1106.4 ± 234	0.168
Minute 3	940.4 ± 210	1050.0 ± 166	0.237
**RMSSD in milliseconds**			
Baseline	68.4 ± 36.4	73.9 ± 65.5	0.594
Minute 1	144.6 ± 108.0	151.7 ± 104.1	0.929
Minute 2	106.9 ± 78.1	171.8 ± 98.4	0.114
Minute 3	131.6 ± 50.4	134.6 ± 82.3	0.790
**HF in milliseconds**^**2**^			
Baseline	1,491.1 ± 1,190	2,032.8 ± 2,446	0.910
Minute 1	12,417.6 ± 11,001	9,105.6 ± 10,814	0.213
Minute 2	5,633.5 ± 6,009	10,155.2 ± 9,184	0.263
Minute 3	6,056.9 ± 7,455	5,864.9 ± 5,612	0.965
**LF:HF ratio**			
Baseline	0.65 ± 0.40	0.99 ± 0.63	0.422
Minute 1	0.88 ± 0.41	0.62 ± 0.85	**0.050**
Minute 2	0.98 ± 0.84	0.51 ± 0.72	0.094
Minute 3	0.41 ± 0.21	0.58 ± 0.46	0.594

*Data are presented as mean ± standard deviations. Bold values indicate statistical significance*.

**Figure 1:**
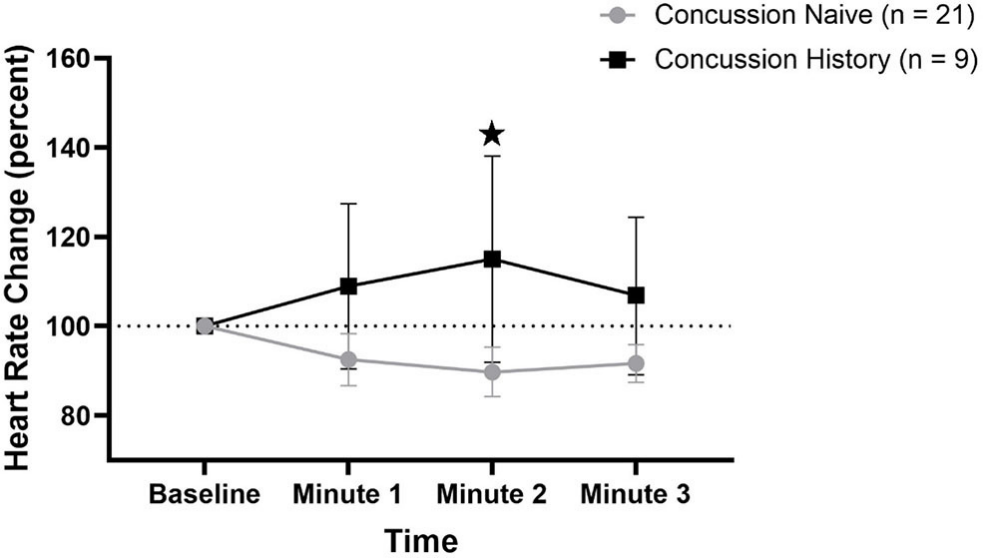
HR percent change during FC with 95% CI. * indicating significant difference between groups on repeated measures.

HR percent change from baseline data are presented in Figure 1. There was a difference over time between groups (*p* = 0.021) that was not affected by sex (*p* = 0.792) or age (*p* = 0.097). At minute 1, CH had a mean change of +8.9% (−9.6, +27.4) while CN had a mean change of −7.5% (−13.3, −1.7). At minute 2, CH had a mean change of +15.0% (−8.0, +38.1) while CN had a mean change of −10.3% (−15.8, −4.7). At minute 3, CH had a mean change of +6.9% (−10.1, +24.4) while CN had a mean change of −8.3% (−12.6, −4.1).

**Figure 2:**
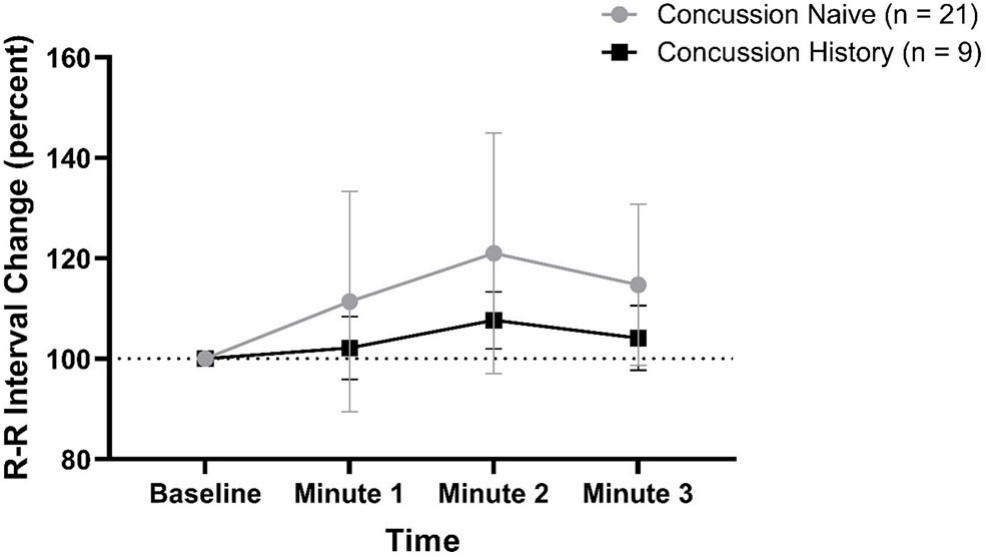
RRI percent change during FC with 95% CI.

RRI percent change from baseline data are presented in Figure 2. There was no difference over time between groups (*p* = 0.161) and there was no effect of sex (*p* = 0.582) or age (*p* = 0.385). At minute 1, CH had a mean change of +2.2% (−4.0, +8.4) while CN had a mean change of +11.4% (−10.5, +33.4). At minute 2, CH had a mean change of +7.7% (+2.0, +13.4) while CN had a mean change of +21.0% (−3.0, +44.9). At minute 3, CH had a mean change of +4.2% (−2.3, +10.6) while CN had a mean change of +14.7% (−1.3, +30.8).

**Figure 3:**
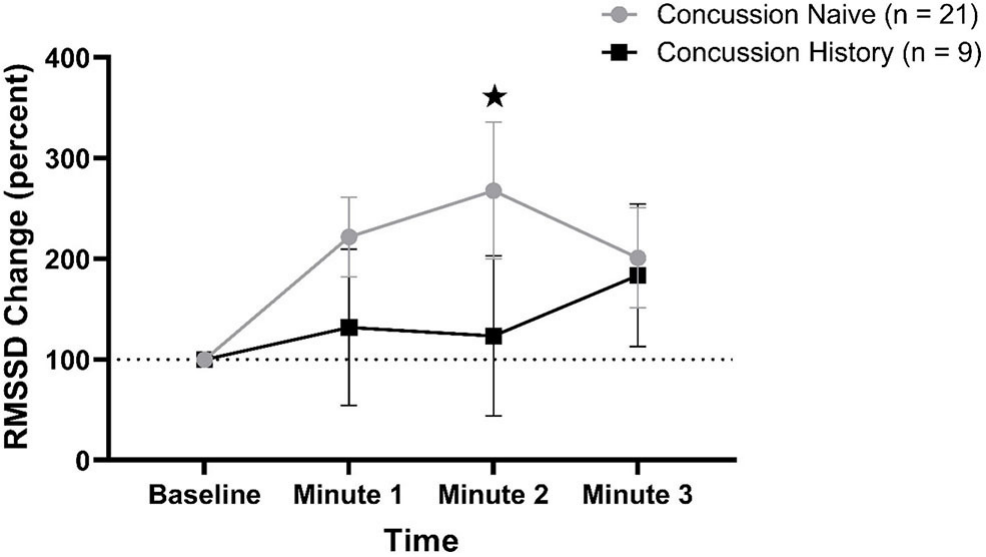
RMSSD percent change during FC with 95% CI. * indicating significant difference between groups on repeated measures.

RMSSD percent change from baseline data are presented in Figure 3. There was a difference over time between groups (*p* = 0.048) that was not affected by sex (*p* = 0.084) or age (*p* = 0.597). At minute 1, CH had a mean change of +31.8% (−44.8, +109.4) while CN had a mean change of +121.8% (+82.1, +161.5). At minute 2, CH had a mean change of +23.4% (−56.1, +102.8) while CN had a mean change of +167.7% (+88.7, +235.5). At minute 3, CH had a mean change of +83.6% (+12.9, +154.2) while CN had a mean change of +100.9% (+51.2, +150.7).

**Figure 4:**
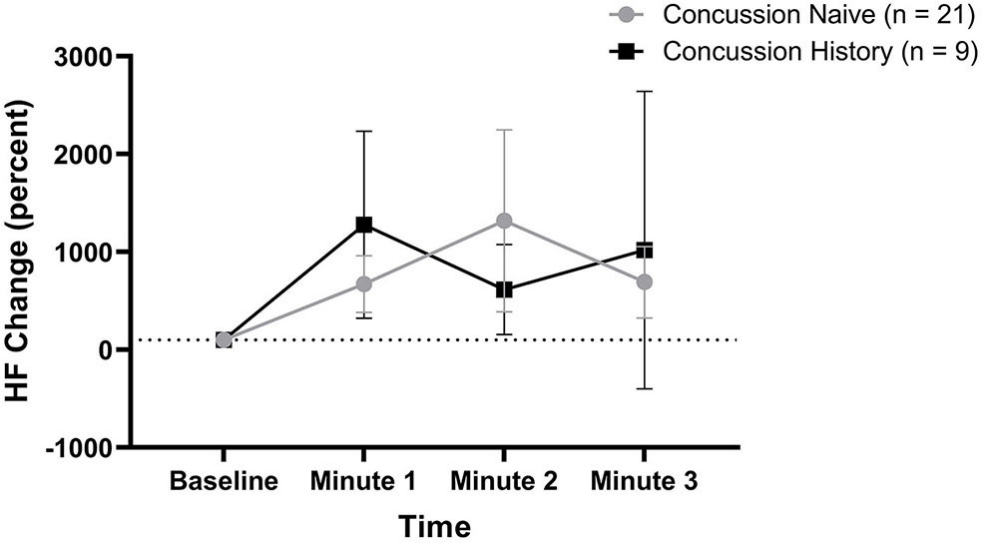
HF percent change during FC with 95% CI.

HF percent change from baseline data are presented in Figure 4. There was no difference over time between groups (*p* = 0.550) and there was no effect of sex (*p* = 0.275) or age (*p* = 0.660). At minute 1, CH had a mean change of +1,177% (+221, +2,134) while CN had a mean change of +571% (+282, +861). At minute 2, CH had a mean change of +514% (+54, +975) while CN had a mean change of +1,217% (+286, +2,148). At minute 3, CH had a mean change of +1,019% (−500, +2,540) while CN had a mean change of +593% (+234, +952).

**Figure 5:**
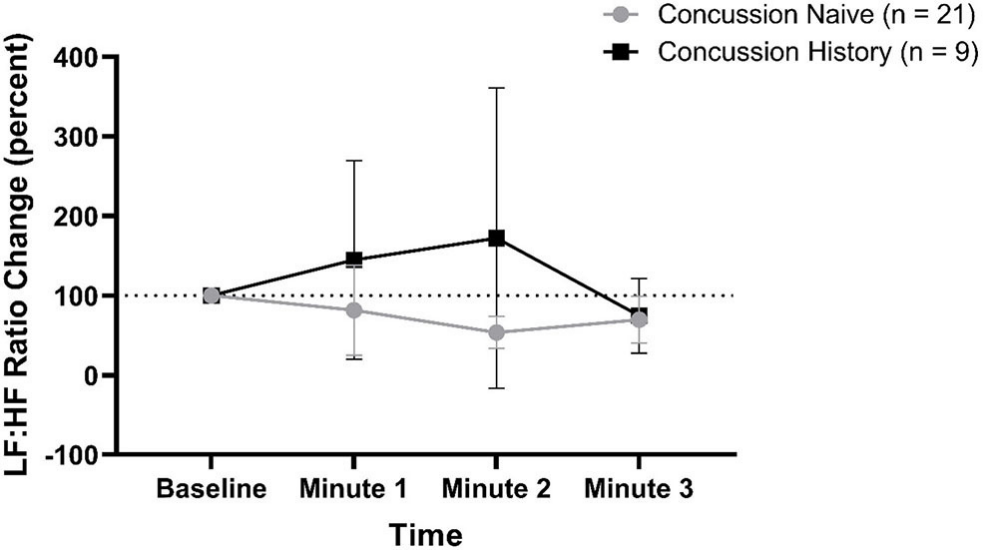
LF:HF ratio percent change during FC with 95% CI.

LF:HF percent change from baseline is presented in Figure 5. There was no difference over time between groups (*p* = 0.062) and there was no effect of sex (*p* = 0.288) or age (*p* = 0.956). At minute 1, CH had a mean change of +44.7% (−79.9, +169.3) while CN had a mean change of −18.5% (−75.2, +38.2). At minute 2, CH had a mean change of +72.0% (−116.9, +261.0) while CN had a mean change of −46.4% (−66.5, −26.4). At minute 3, CH had a mean change of −25.4% (−73.3, +21.7) while CN had a mean change of −30.3% (−59.8, −0.8).

## Discussion

This pilot investigation shows that student athletes who reported experiencing a concussion more than a year ago had a blunted cardiac parasympathetic response to FC vs. student athletes who reported never having had a physician-diagnosed concussion. Participants without a history of concussion demonstrated the typical increase in RMSSD from baseline in response to stimulation of the trigeminal nerve with ice water, which is an indirect measure of cardiac parasympathetic activity (28). Participants who reported having a concussion more than a year ago, however, had a blunted response during the first 2 min of FC that was equivalent to the response we found in acutely (<10 days since injury) concussed college (14) and high school (19) athletes in previous studies. Heart rate in CH group increased from baseline to the end of minute 1 during FC, which is the opposite of the reduction in HR typically seen with cold stimulation of the trigeminal nerve. These data suggest that cardiac parasympathetic activity did not predominate during FC as it should in CH group, which supports the concept that athletes with prior concussions have difficulty “switching” to the appropriate branch of the ANS in response to environmental stimuli (29). CH had a significantly lower resting HR at baseline than CN yet had almost identical absolute HRs at minute 1 of FC; hence, the significant difference in change from baseline may reflect this difference in resting HR. Mean HR, however, continued to increase in CH from minutes 1 to 2 during FC whereas it declined in CN, which is consistent with normal parasympathetic function. HR change, LF:HF change and RMSSD change returned to normal by minute 3, which was expected due to engagement of the sympathetic response typically seen by minute 3 with continued FC (13). We did not identify significant changes in HF power between CN and CH. Although HF is thought to represent parasympathetic activity, it is commonly called the “respiratory frequency” because it corresponds to the HR variations related to the respiratory cycle (28). It is not considered, however, to provide additional information beyond time-domain measures (such as RMSSD) for vagal parasympathetic activity (27, 28). Since we did not collect respiratory data in this study, the HF data are difficult to interpret. Future studies in this realm should collect respiratory data to be able to interpret HF responses.

Other investigators have demonstrated persistent sympathetic ANS dysfunction in concussed athletes. Abaji et al. (17) reported that concussed patients in the post-acute to late phase after injury (mean 95 ± 63 days) had a blunted HRV response to isometric handgrip exercise (IHGE) vs. healthy controls. La Fountaine et al. (18) reported a blunted cardiac autonomic response during IHGE within 2 weeks after concussive head injury that was not present at rest. These studies may not, however, directly relate to our study since IHGE is a sympathetic stimulus that does not engage cardiac parasympathetic activity (30). Our data reveal that cardiac parasympathetic dysfunction persists for far longer beyond clinical recovery than previously shown in athletes after SRC.

Emerging research implicates ANS dysfunction as one cause of concussion signs and symptoms, including exercise intolerance (31), vision problems (32), and anxiety (33). Our data suggest, however, that the autonomic cardiac response to a stressor remains impaired in athletes with remote prior concussions who are not reporting any concussion-like symptoms at rest or during physical exertion. Our participants were doing well in school and playing organized sports without limitation. We must consider the possibility that the blunted autonomic response we measured over a year after recovering from a concussion is somehow mitigated by other mechanisms so that the body is able to function in this state without overt concussion-like symptoms or impairments. It is currently unclear if persistent autonomic dysfunction is a contributing factor to the increased susceptibility to repeat concussion in those who have had one or more concussions (34). Data from our study have relevance to future concussion research. Studies assessing physiological function after concussion may want to consider enrolling control participants with no lifetime history of concussion to reduce the possibility that previously concussed (but currently asymptomatic) athletes have ongoing sub-clinical physiological dysfunction. This would aid researchers in developing diagnostic biomarkers with improved specificity for identifying acute concussion and for more objectively determining recovery.

## Limitations

The major limitation of this study is the small sample size with unbalanced groups. This study was a retrospective analysis of participants who were recruited as healthy non-concussed controls in prospective case-control studies; hence, this convenience sample may not be representative of the general student-athlete population. We did not collect comprehensive details of previous concussions, such as loss of consciousness, recovery time or sport/activity. Future studies should obtain a detailed concussion history, including a history of concussion-like events that were never diagnosed by a physician/clinician, although it is recognized that self-reported concussion history may not be reliable (35). Future studies should also assess duration of participation and position played to explore a potential relationship between possible “sub-concussive” head impacts and ANS responses. Our study did not account for several variables that can affect HRV, including anxiety, variation in circadian rhythm patterns, sleep, endocrine factors and respiration (25). Future studies should attempt to control for these factors by either standardizing the time slept the night prior, performing the test soon after awakening or standardizing time since awakening to control for variation in circadian and endocrine cycles, measuring respiration and end-tidal CO_2_ during the FC test, and performing more than one test to assess reliability. Finally, prospective longitudinal studies with multiple timepoints are needed to identify when, or if, impaired autonomic function returns to normal or baseline values after SRC.

## Conclusion

Our data show that athletes with a remote history of concussion who are currently participating in sports and school without limitation have a blunted response to an effort-independent test of cardiac parasympathetic function. These results add to emerging data that physiological disturbances persist despite clinically-determined recovery from SRC. Our data suggest that cardiac autonomic dysfunction may persist for longer than expected. This has implications for the design of future concussion physiology studies, for susceptibility to repeat concussion, and potentially for finding a more objective determination of SRC recovery and readiness to return to sport.

## Data Availability Statement

The raw data supporting the conclusions of this article will be made available by the authors, without undue reservation.

## Ethics Statement

The studies involving human participants were reviewed and approved by University at Buffalo Institutional Review Board. Written informed consent to participate in this study was provided by the participants' legal guardian/next of kin.

## Author Contributions

MH, BJ, JL, and BW contributed to study conception and design, interpretation of the results, statistical analysis, and manuscript writing. EH, CW, ER, MO'L, AB and LD contributed to participant enrollment, conducting experiments, data collection and preprocessing, and data quality assessment. All authors contributed to the article and approved the submitted version.

## Conflict of Interest

The authors declare that the research was conducted in the absence of any commercial or financial relationships that could be construed as a potential conflict of interest.
